# A Synthetic Biology Approach for Vaccine Candidate Design against Delta Strain of SARS-CoV-2 Revealed Disruption of Favored Codon Pair as a Better Strategy over Using Rare Codons

**DOI:** 10.3390/vaccines11020487

**Published:** 2023-02-20

**Authors:** Pankaj Gurjar, Noushad Karuvantevida, Igor Vladimirovich Rzhepakovsky, Azmat Ali Khan, Rekha Khandia

**Affiliations:** 1 Department of Science and Engineering, Novel Global Community Educational Foundation, Hebersham, NSW 2770, Australia; 2 College of Medicine, Mohammed Bin Rashid University of Medicine and Health Sciences, Dubai P.O. Box 505055, United Arab Emirates; 3 Medical and Biological Faculty, North Caucasus Federal University, 355017 Stavropol, Russia; 4 Pharmaceutical Biotechnology Laboratory, Department of Pharmaceutical Chemistry, College of Pharmacy, King Saud University, Riyadh 11451, Saudi Arabia; 5 Department of Biochemistry and Genetics, Barkatullah Universty, Bhopal 462026, India

**Keywords:** synthetic biology, SARS-CoV-2 delta vaccine candidate, codon optimization, rare codon, codon pair, codon context

## Abstract

The SARS-CoV-2 delta variant (B.1.617.2) appeared for the first time in December 2020 and later spread worldwide. Currently available vaccines are not so efficacious in curbing the viral pathogenesis of the delta strain of COVID; therefore, the development of a safe and effective vaccine is required. In the present study, we envisaged molecular patterns in the structural genes’ spike, nucleoprotein, membrane, and envelope of the SARS-CoV-2 delta variant. The study was based on determining compositional features, dinucleotide odds ratio, synonymous codon usage, positive and negative codon contexts, rare codons, and insight into relatedness between the human host isoacceptor tRNA and preferred codons from the structural genes. We found specific patterns, including a significant abundance of T nucleotide over all other three nucleotides. The underrepresentation of GpA, GpG, CpC, and CpG dinucleotides and the overrepresentation of TpT, ApA, CpT, and TpG were observed. A preference towards ACT- (Thr), AAT- (Asn), TTT- (Phe), and TTG- (Leu) initiated codons and aversion towards CGG (Arg), CCG (Pro), and CAC (His) was present in the structural genes of the delta strain. The interaction between the host tRNA pool and preferred codons of the envisaged structural genes revealed that the virus preferred the codons for those suboptimal numbers of isoacceptor tRNA were present. We see this as a strategy adapted by the virus to keep the translation rate low to facilitate the correct folding of viral proteins. The information generated in the study helps design the attenuated vaccine candidate against the SARS-CoV-2 delta variant using a synthetic biology approach. Three strategies were tested: changing TpT to TpA, introducing rare codons, and disrupting favored codons. It found that disrupting favored codons is a better approach to reducing virus fitness and attenuating SARS-CoV-2 delta strain using structural genes.

## 1. Introduction

Classically the live attenuated vaccine candidates are prepared through serially passaging it in non-optimal conditions, which leads to the attenuation of the virus [1]. Vaccine candidate generation through this method is a lengthy and stochastic process. For human poliovirus, the Sabin vaccine, and for the Rinderpest virus, the Plowright vaccine has been developed. Some key mutations are responsible for virus attenuation. However, during serial passages, reversion to the wild-type phenotype through reversion in those small number of key attenuation mutations is possible, resulting in loss of attenuation and remains the major problem with such vaccine candidates [2]. It has been documented in the Polio virus [3], Infectious Bursal Disease virus [4], Canine Distemper Virus [5], and highly pathogenic Porcine Reproductive and Respiratory Syndrome virus [6] also. Novel strategies are based on introducing a large number of mutations that individually impart little role in reducing the replicative fitness but cumulatively generate significant attenuation with relatively high genetic stability [7,8].

Two or more than two codons code an amino acid called synonymous codons. These codons are not used equally, and unequal usage is referred to as codon bias. This biological phenomenon of codon bias may be used for both codon optimization (CO) and deoptimization (CD). The CO, on the one hand where optimal codons are used; on the other hand, in CD, original codons are replaced with less-preferred codons [9]. The feasibility of generation of attenuated viruses by codon deoptimization has been shown in the influenza A virus [10], arenaviruses lymphocytic choriomeningitis virus [11], Lassa virus [12], ΦX174 [13], respiratory syncytial virus [14], and human immunodeficiency virus type 1 virus [15]. Contrarily, despite enhancing protein production, adenovirus fiber protein codon optimization resulted in virus attenuation [16]. Similarly, in RSV, lowered replicative ability of codon-optimized virus has been observed in mice [14].

The goal of recoding the virus is to modify the dinucleotide, codon, or codon pair composition of the recoded viral genes to produce a replication-competent but attenuated vaccine candidate. Hundreds of mutations are generated during the recoding of a virus, but amino acid composition remains the same. Hence recoded viruses antigenically remain similar to their wild-type parents [17].

Like codon bias, there is a bias present in two adjacent codons also, which is called codon pair bias (CPB). Codon pairs impact gene expression, and altering the codon pairs towards the codon pairs which are disfavored by the host or virus itself has been recently used as a strategy to reduce the replicative virus fitness. The method can produce a new generation of safer, non-reverting, live attenuated vaccines. Selection for disfavoured CPs results in unintended increases in CpG and UpA dinucleotide frequencies [18,19]; those are the target of zinc finger antiviral protein and RNAseL that directly binds to the high CpG RNA sequences and contribute to virus attenuation [20]. The presence of a large number of underrepresented codons interferes with protein production or processing, and possibly physical properties of specific tRNAs, including 3D structures, hamper the optimal fitting into adjacent aminoacyl- and peptidyl-sites in the translating ribosome [21].

Alpha, Beta, Gamma, Delta, Epsilon, Iota, Kappa, Lambda, and Omicron variants of SARS-CoV-2 are present. Of these, Alpha, Beta, Gamma, Delta, and Omicron were declared as variants of concern (VoCs) by the WHO [22]. India experienced a sudden rise in COVID-19 cases since late march 2021, causing more than 400,000 cases and 4000 deaths reported each day in early May 2021. The B.1.617.2 (delta) variant was detected for the first time in India in December 2020 and later became the most commonly reported variant across the globe [23], and it was associated with global surges in cases, higher viral loads, longer duration of infectiousness, and high rates of reinfection [24]. Genome sequencing data also revealed that novel variants caused breakthrough cases, such as alpha (B.1.1.7, 56%), epsilon (B.1.429, 25%), B.1.427 (8%), gamma (P.1, 8%), and beta (B.1.351, 4%) [25], accounting for 56%, 25%, 8%, 8%, and 4% of breakthrough infection. Contrarily 86.69% of breakthrough was due to delta [26], pointing to its greater involvement. In vaccinated and unvaccinated people, the severity of disease on the WHO clinical progression scale was highest for the delta group, followed by alpha, and least for omicron among adults admitted to hospitals in the United States [27]. In addition, delta strain infection demanded more oxygen therapy than Alpha or Omicron [28]. Higher viral loads, longer duration of infectiousness, high disease severity and requirement of hospital admission and oxygen therapy, and high rates of post-infection breakthrough prompted authors to investigate vaccine candidate development against the Delta strain.

Vaccines are reported to be efficacious against infectious diseases, as shown by clinical trials [29]. The data suggested that the vaccination gave protection against severe disease outcomes in the case of the B.1.1.7 (alpha) variant, isolated first in the United Kingdom [30]. For the B.1.351 (beta) variant, effectiveness against the severe disease was reported to be low; however, it still reduced severe and fatal outcomes in individuals vaccinated with the BNT162b2 vaccine [31]. The same BNT162b2 vaccine exhibited a high level of neutralization against the P.1 (gamma) variant [32].

Delta variant possesses mutations in the spike region of the virus (spike protein mutations T19R, Δ157-158, L452R, T478K, D614G, P681R, and D950N). These mutations enable the delta strain to escape the immune response. The data is limited on protection by vaccination with BNT162b2 and ChAdOx1 nCoV-19 against symptomatic delta strain. David W. Eyre (2022) [33] reported that two vaccinations with either BNT162b2 or ChAdOx1 nCoV-19 caused a small reduction in delta variant transmission than the alpha variant, as evidenced by a partial reduction in the PCR Ct values. The effectiveness of the BNT162b2 vaccine against symptomatic COVID-19 was 57% after the first vaccine dose in adolescents [34].

Furthermore, Luo CH and Morris CP (2021) [35] reported the delta variant as a cause of higher infectious virus loads in both vaccinated and unvaccinated individuals. Considering the partial effectiveness of available vaccines against the delta strain, it is essential to design strategies that effectively target the delta variant. In the present study, we attempted to gain insights into molecular patterns present in the structural genes (spike (S), nucleoprotein (N), membrane (M), and envelope (E)) of the COVID delta strain, which can be explored into the synthetic biology approach to develop a vaccine candidate.

## 2. Materials and Methods

### 2.1. Sequence Retrieval

We retrieved the sequences from the National Center for Biotechnological Information (NCBI) for the SARS-CoV-2 delta strain. The sequences taken were collected between January 2022 to July 2022. SARS-CoV-2 is the plus-sense, single-stranded viral RNA genome that encodes open-reading-frames (ORFs) for sixteen non-structural proteins that form the replication machinery (ORF1a/ORF1b), four structural proteins (spike (S), nucleoprotein (N), membrane (M) and envelope E)), and seven accessory proteins. Both the structural and non-structural proteins can be targeted for virus attenuation. Still, we chose to focus on structural proteins since these proteins are essential for the host cells’ binding and invasion, and immune response against them will be able to provide an effective barrier against viral binding and invasion in the host cell.

A total of 190 sequences for each structural gene encoding for the spike, nucleoprotein, membrane, and envelope were obtained. All selected sequences did not contain any ambiguous sequences, started with ATG and ended with stop codons TAA, TAG, or TGA, and were present in a triplet (The accession numbers of the sequences are given in Supplementary Table S1). For the convenience of study for recoding of envisaged genes, we took the E, M, NP and S genes from the delta virus strain (assession numberOM982659.1). Sequences for the E, M, N, and S genes of representative sarbecoviruses were taken from the work of Llanes et al. (2020) [36] in the study that included Bat SARS-like CoV RaTG13 (MN996532), Bat SARS-like CoV HKU3 (DQ022305), Bat SARS-like CoV SL-CoVZC45 (MG772933), Bat SARS-like CoV SL-CoVZXC21 (MG772934), Bat SARS-like CoV WIV1 (KF367457), SARS-CoV (Human, NC_004718), SARS-CoV (Civet, AY686863), SARS-CoV-2 (Human, NC_045512), SARS-CoV-2 (Tiger, MT365033), and Pangolin CoV (MT040333). Representative sequences from VOCs Alpha (MZ622337), Beta (MZ344999), Gamma (MZ477758), and Omicron (OQ084152) were also included.

### 2.2. Odds Ratio Analysis

The odds ratio is expected to observe dinucleotide frequency in a given nucleotide sequence. Various factors affect the odds ratio, including nucleotide composition [37], evolutionary forces [38], forces required to maintain RNA secondary structures involved in splicing and gene expression [39], and forces to evade host defense mechanism (specific context is CpG where viral pathogens avoid CpG since CpG dinucleotide are perceived as pathogen-associated molecular patterns by host cells, and viral pathogens tend to decrease CpG content [40]. For the four genes, the dinucleotide odds ratio was calculated using DNASTAR Lasergene Inc. An odds ratio value of 0.78 and 1.23 is considered underrepresentation and overrepresentation of dinucleotides, respectively [41].

### 2.3. Relative Synonymous Codon Usage (RSCU) Analysis

RSCU help in knowing the preferred and non-preferred codons. The relative synonymous codon usage (RSCU) is calculated using the formula.

RSCU = S × Nc/Na

where S = the number of synonymous codons encoding the same amino acid.

Nc = the frequency of the codon in the genome.

Na is the relative frequency of the codon for that amino acid.

### 2.4. Codon Context Analysis

The effects of adjacent sequences on protein translation are called context effects. There are experimental pieces of evidence suggestive of the effects of context on nonsense suppression, missense suppression, translational errors, and frameshifting, which is further supported by statistical analysis that explain that the context around codons is not random [42]. Also, the codon context affects translational kinetics [43]. Codon context analysis was done using Anaconda software 2^®^ [44]. The context was evaluated into the matrix of 64 × 64, where stop codons were included, and the direction was kept 5′ to 3′.

### 2.5. High Occurring Codon Pairs

The efficient translation is dependent on the usage of codons and codon pairs. Some synonymous codons are used more in comparison to other codons, which is called codon bias. Similarly, some codon pairs are also frequently used and referred to as codon pair bias. An example is codon pair GCA-GAG, which is preferentially used to encode amino acid pair alanine-glutamic acid compared to GCC-GAA [45]. Both codon deoptimization and codon pair deoptimization are used to attenuate viruses [9,10,46]. High-occurring codon pairs were determined by Anaconda 2.0 version assessed on 24 July 2022. The generated report was trimmed, and the top 20 high-occurring codon pairs were taken.

### 2.6. Rare Codon Analysis

The usage of rare codons in the reading frame is used to control the translation rates and adopt an intermediate confirmation to attain proper protein folding [47]. In a few instances, substituting rare codons with the optimal one resulted in protein misfolding and affecting solubility [48] and, eventually, loss of biological activity [49]. The presence of rare codons might be tissue-specific [50] and indicative of translational programming of cell proliferation [51]. The number of rare codons was calculated for all 4 genes and normalized to get percent occurrence. An occurrence of less than 0.5% was set as a criterion to be rare codons, and above than 5% was considered abundant codons.

### 2.7. Codon Pair Score

Codon pair bias can be quantified using the codon pair score (CPS) statistics [45]. The codon pair bias (CPB) indicates the bias present in the codon pair, and it is the mean of the codon pair scores (CPSs) for all of its codon pairs present in an ORF or gene. In turn, the CPS for each codon pair is the natural log of the ratio of the observed versus expected frequency of that codon pair [52]. Statistically, underrepresented codon pairs have negative, and overrepresented codon pairs have positive CPS values. The average CPS of a gene is calculated as the arithmetic mean of individual CPS values. The lower the value of CPS, the more the virus will be attenuated.

### 2.8. mRNA Stability Calculation

mRNA stability is important in regulating protein expression [53]. Genome-wide RNA decay analysis revealed that stable mRNA are generally rich in optimal codons and result in high gene expression, while unstable mRNA encompass predominately non-optimal codons. In unstable RNAs, more than 60% of the codons are non-optimal [54]. The higher the stability of an mRNA in the cytoplasm, the higher quantities of proteins will be produced. Several algorithms like mfold, KineFold, and ViennaRNA are used to predict plausible mRNA structures. The software computes the mRNA thermodynamic stability value in the form of minimum free energy (MFE), a thermodynamic energy measurement based on intramolecular stacking, the system’s temperature, entropy, enthalpy, and ionic conditions, and hydrogen bond interactions [55]. A lower MFE depicts more stable mRNA [56], and unstable mRNA structures have more than 60% non-optimal codons [54]. Less stable transcripts will have fewer negative values, and with more negative values, better-fit progeny will be generated. The RNAfold server was used to calculate the transcript’s minimum free energy (MFE). 

### 2.9. Codon Adaptation Index (CAI) Calculation

CAI is a common evaluation measure of protein expression [57]. CAI alone is not very comprehensive but imperative to determine gene expression [58]. CAI values were calculated using the CAICal served developed by Puigbò and colleagues [57].

## 3. Results

### 3.1. Compositional Features of SARS-CoV-2 Delta Strain Structural Genes Revealed at Richness

The nucleotide composition of any genome is responsible for mutational robustness, which indicates the capacity to withstand mutations exhibiting no or slight variation in phenotype upon introducing mutations [59] and influence codon usage [60]. Disproportionate base composition accounts for much of codon usage in RNA viruses [61]. In the present study, the structural genes of the delta strain of the SARS-CoV-2 virus were studied for their nucleotide composition (Table 1). The average nucleotide composition of the gene indicated that the genes were AT-rich, with an abundance of T nucleotide (except for the M gene); however, at the third codon position, all the genes have richness in T nucleotide.

### 3.2. Odds Ratio Analysis Indicated Both under and Overrepresentation of Some Mirror Dinucleotides

The odds ratio analysis revealed that TpT and CpA showed maximum variation in values with standard deviations of 0.63 and 0.51, respectively. The average TpT dinucleotide value ranged between 0.867–2.383, while CpA ranged between 0.359–1.569 for the four structural genes of the delta virus. Since the variation was in higher ranges, the deviation was high.

**Figure 1:**
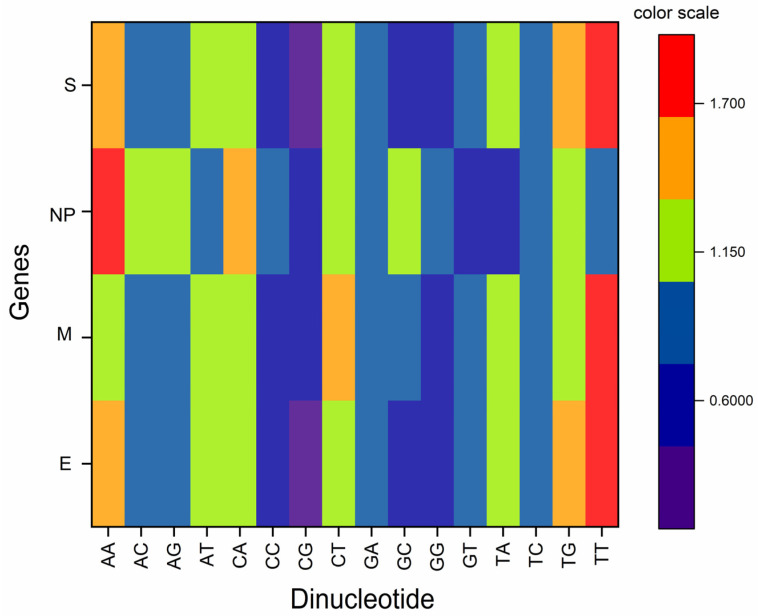
Dinucleotide odds ratio analysis for structural genes of delta strain of SARS-CoV-2.

The least deviation was observed for TpG, ApC, and ApG dinucleotides. TpT dinucleotide was underrepresented in the NP gene, while in other genes, it is overrepresented. CpG, as expected, was underrepresented in all the genes. Based on the average odds ratio, it was evident that GpA, GpG, CpC, and CpG dinucleotides were underrepresented (odds ratio < 0.78), while TpT, ApA, CpT, and TpG were overrepresented (odds ratio > 1.23). Here CpC and GpG and; TpT and ApA are the mirror dinucleotides that are underrepresented and overrepresented, respectively. From the figure, it is evident that the odds ratio of E, M and NP genes are somewhat similar, while for the NP gene, there is little difference (Figure 1).

### 3.3. Dinucleotide Bias at the Junction of Codons

**Figure 2:**
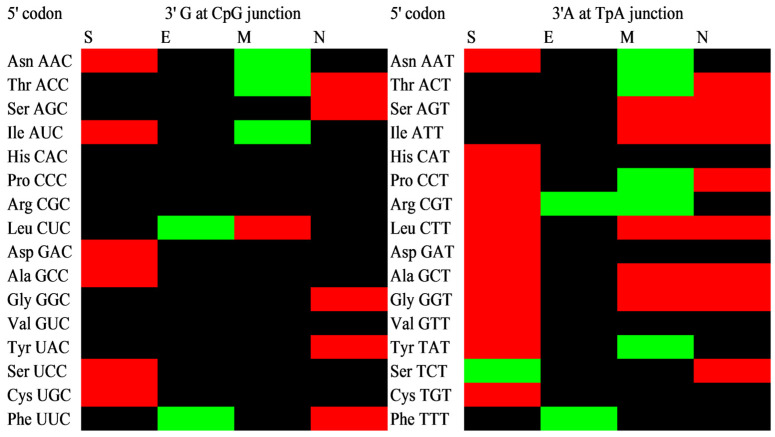
Dinucleotide bias context analysis of CpG and TpA dinucleotide at p3-1 junction. Positive context has been demonstrated with green color, while negative context has been demonstrated with red. Insignificant context (between 15 and −15) is depicted as black color.

Dinucleotide bias at the junction of codons (C at 3rd position of 5′ codon and G at 1st codon position in subsequent 3′ codon and similarly T at 3rd position of 5′ codon and A at 1st codon position in subsequent 3′ codon, is called p3-1 junction), were evaluated for CpG and TpA and demonstrated in Figure 2. Positive and negative contexts were found for CpG and TpA at the p3-1 junction, though the negative context was more prominent than the positive one (Figure 2). The negative context for CpG at the junction was mainly present in the S and N genes. In the E gene, only positive, while in the M gene, both positive and negative contexts were present depending on the amino acid. For TpA dinucleotide at the junction, in gene S, a highly negative context was present, followed by M and N genes. Similar to the CpG junction, the TpA junction was present in a positive context only in the E gene. Overall analysis revealed that at the junction, all kinds of contexts (no context, positive context, negative context) were present; however, in the S gene, negative contexts were more prominent for the TpA junction, which could be the result of selection forces. Our results concord with the results obtained by Beutler et al., 1989 [62]. 

A similar result was obtained on Ustilago, a fungal parasite of grasses, where extensive codon context analysis revealed avoidance of TpA at codon–codon junctions, and possibly it is attributed to reducing the risk of nonsense mutations resulting in a stop codon and abrupt chain termination [63] and affecting subsequent translation fitness as a part of the selection [64]. In contrast to our result, TpA was the second most abundant dinucleotide at the junction in Human Rhinoviruses A, B, and C [65]. CpG frequency is dropped in the Influenza A virus at the p3-1 junction [66], and it is additional to the intracodon CpG component, where all CpG-containing codons were underrepresented [67]. 

TpA and CpG underrepresentation at the p3-1 junction suggested that codon choices alone may not explain the scarcity of TpA and CpG since, at this position, at least TpA has no defined coding function in this frame and is the result of multiple forces, including immune pressure [67,68], high mutability resulting in a transition from CpG to TpG [69], selection forces [70], TpA having mRNA destabilizing effect [62], higher susceptibility of UpA to cytoplasmic RNase [71] and evading interferon-inducible protein ZAP and RNAseL as host protein responsible for sensing CpG in viral RNA. 

### 3.4. RSCU Values of Codons from Four Structural Genes Revealed That for All Genes; Preferred Codons Are Not the Same

Relative synonymous codon usage (RSCU) is one of the imperative parameters for evaluating the codon bias present in synonymous codons. It represents the expected occurrence frequency of any codon out of all synonymous codons for a particular amino acid, multiplied by the degeneracy level and suggestive of codon priority among synonymous codons encoding for a single amino acid [72]. A higher RSCU values suggest a preferred codon, while lower values are indicative usage of non-preferred codons. 

The RSCU values below 0.6 suggest low occurrence, while values above 1.6 suggest vice versa. CpG and TpA suppression is expected owing to facts mentioned in this article in the above section and observed in vertebrate viruses also [73]. CpG and TpA dinucleotide suppression is reflected in the CpG and TpA encompassing codons, and the same is evidenced by RSCU analysis of codons in various virus models. All eight CpG-encompassing codons are found to be underrepresented in the Nipah virus [74]. RSCU values analysis of six codons containing TpA (TTA, CTA, ATA, GTA, TAT, and TAC) indicated that these are not preferred in Mycoviral genes [75]. HCV also showed a significant tendency to not prefer the codons with CpG or TpA dinucleotides [76], and many researchers have reported similar results [77,78,79,80] establishing a correlation between the presence of CpG and TpA and lower RSCU.

The RSCU value is independent of the amino acid composition of any gene and hence helps compare different genes [81]. Values near 1 suggest the unbiased use of codons [82]. In a synthetic recoded virus vaccine candidate construct, the higher RSCU values codons must be replaced with the lower RSCU valued codon. The RSCU values for each of the genes envisaged and given in Table 2 below. The analysis indicated that each gene codon usage pattern is different.

### 3.5. Codon Usage Comparison for Other Variants of Concern (VOCs) of SARS-CoV-2 and Representative Sarbecoviruses

The average RSCU value of SARS-CoV-2 VoCs and representative Sarbecoviruses is given in Table 3 and compared with delta strain. The analysis revealed that though the RSCU values for each codon slightly differed for different strains, the preferred codon choice remained the same for all amino acids in all envisaged viruses excluding phenyl alanine. 

### 3.6. ACT-, AAT-, TTT- and TTG-Initiated Codons Were Preferred in at Least Three out of Four Genes

Codon pair deoptimization (CPD) is an efficient virus attenuation technique where suboptimal pair of codons is used, and synonymous codons are changed so that amino acid composition remains the same and hence the antigenicity [7]. The ultimate goal of codon swapping is to increase the number of underrepresented codon pairs in the virus’s genes. The strategy has been implicated in attenuating viruses for making vaccine candidates, including human respiratory syncytial virus [83], porcine reproductive and respiratory syndrome viruses [84], enterovirus A71 [85], and dengue virus 2 [86], and the list is long. In the present study, we presented both the high occurring codon pairs and low-occurring codons so that the high-occurring pairs may be disrupted with the low-occurring codons. Table 4 presents the top 20 most preferred codon pairs in the envisaged genes. Analysis revealed that among the top 20 most preferred codon pairs, ACT- (Threonine) AAT- (Asparagine), TTT- (Phenyl alanine) and TTG- (Leucine) initiated codons were preferred in at least three genes out of four envisaged. On the other hand, GTT-, GGA- and CTT- initiated (Val, Gly and Leu) codons were preferred in at least two genes.

### 3.7. Preferred Codon Pair Analysis in Sarbecoviruses and Other SARS-CoV-2 VoCs

Delta virus structural genes were compared with the other strains of SRAS-CoV-2 and sarbecoviruses, and the top 20 codon pairs for each of the genes are given in Table 5A–D. When the E gene of all envisaged strains was compared, the analysis revealed that in delta and other strains, Phenylalanine-, Leucine-, Serine-, and Tyrosine-initiated codons are preferred. The difference was in Valine-initiated codons, which were abundant in delta (05 valine-initiated codon pairs), while in other strains of SARS-CoV-2 Serine initiated (06), codon pairs were preferred (Table 5A). For the M gene, all the strains envisaged preferred Phenyl alanine-initiated and Leucine initiated codons. However, the number of Phenyl alanine-initiated codon pairs was less (04) in the delta strain compared to 06 Phenyl alanine-initiated codon pairs in others, including Sarbecoviruses. Also, in delta Leucine-initiated, 05 codon pairs were present, while their number was 07 in other viruses (Table 5B). For the N gene, Glycine-initiated codon pairs (05) were preferred in SARS-CoV-2 VoCs, excluding Sarbecoviruses and delta. In the delta, only 03 codon pairs were Glycine-initiated, while in Sarbecoviruses, Lysine-initiated codon pairs were preferred (04) (Table 5C). For the S gene, the delta strain and other SARS-CoV-2 strains Glycine-initiated (≥03) codon pairs were preferred. In Sarbecoviruses, no such clear pattern was observed. Furthermore, in Omicron, Gycine-initiated (04) codon pairs were preferred (Table 5D). Based on the analysis, it can be said that since the codon preference is the same for all VoCs, including delta and Sarbacoviruses, the choice of the preferred codons is also similar to some extent. However, for codon pairs, the choice differed to some extent when the delta was compared with others. The difference may result from complex molecular interactions or signature molecular patterns. Since we included only the top 20 codon pairs in the study, other shared codon pairs between the viruses are possible. 

### 3.8. Codon Context Revealed Highest Codon Pair Bias in Spike Protein

**Figure 3:**
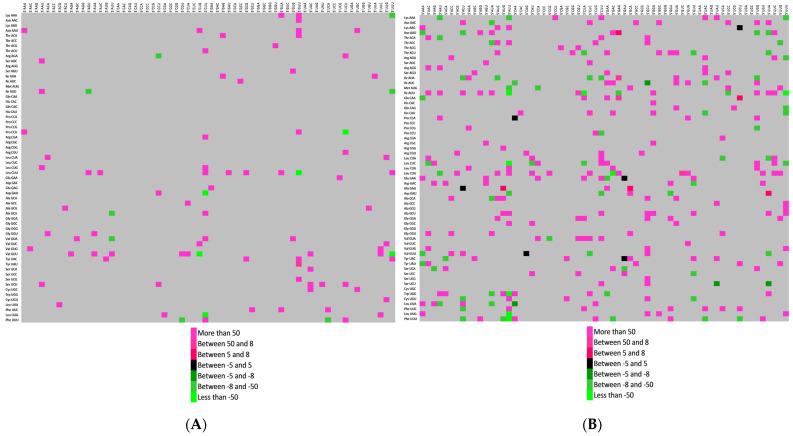

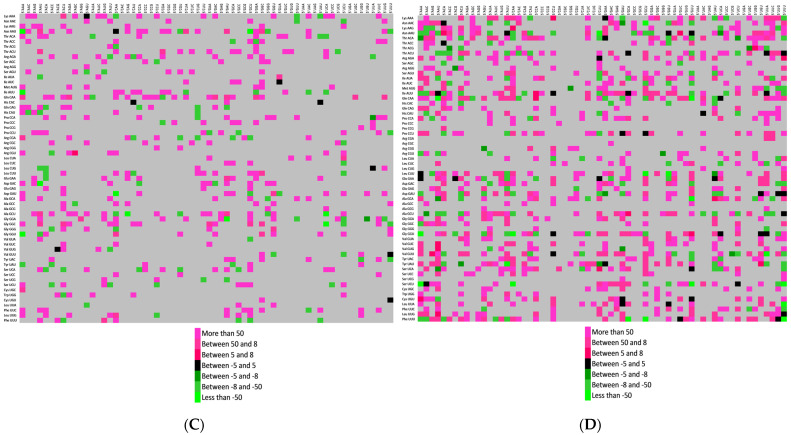
Codon context analysis for the (**A**) E gene, (**B**) M gene, (**C**) NP gene, and (**D**) S gene. The two-color scale shows the codon context bias. Strongly preferred codon context bias is depicted as pink, while strongly rejected codon context is depicted as green. In the case where the 3′ context is not strongly biased, it is depicted as black. The Grey color shows the presence of no context. The color scale is given in the figures.

A substantial bias is present during codon pair utilization, called dicodon bias or codon context. It is a well-recognized phenomenon and is considered to arise from GC-biased gene conversion [87]. It is a direct cause of dinucleotide bias [18]. We performed codon context analysis for four genes of SARS-CoV-2, and all kinds of contexts ((negative (residual values less than −5), positive (residual values more than +5), insignificant (residual values between −5 and 5), and no context (residual zero)) were found in these genes. The insignificant context was absent in the envelope gene, while in the spike gene, the maximum positive and negative codon pair biases were present (Figure 3A–D).

The E, M, N, and S genes encode structural proteins [88]. The S gene plays a crucial role in receptor recognition and cell membrane fusion [89]. The sizes of the E, M, N, and S genes are 228bp, 669bp, 1260bp, and 3822 bp, respectively. Translational selection shapes codon context [90], and nonsense and missense suppression, elongation rate, the precision of tRNA selection and polypeptide chain termination all appeared to be affected by codon context [91]. Since the size of the S gene is the largest among all the envisaged genes, we speculate that the above-stated factors will be more operative on larger genes due to the very nature of the longer gene. Furthermore, at least in our envisaged genes, we found the same pattern, and codon context bias increased with the size of the gene. Therefore, the comparatively larger size of the S gene is attributed to maximum codon context bias, and a more positive context may be a molecular signature of the S gene.

### 3.9. Codons CGG (Arg), CCG (Pro) and CAC (His) Were Rare in All the Genes

Rare codons are not randomly present inside the mRNA sequence, indicating operative selective forces. Rare codons help initiate proper protein folding in nascent peptides and prevent the formation of secondary structures in mRNA in the 5′ region [92]. Like optimal codons, rare codons are also maintained through evolutionary forces. The incorporation of rare codons has been shown to reduce the translation of poliovirus capsid protein resulting in virus attenuation [47]. Using Anaconda2 software, we calculated the number of rare codons and then normalized them with gene length. An occurrence rate below 0.5% was considered a rare gene codon, which is a default value given by Anaconda2 software.

**Figure 4:**
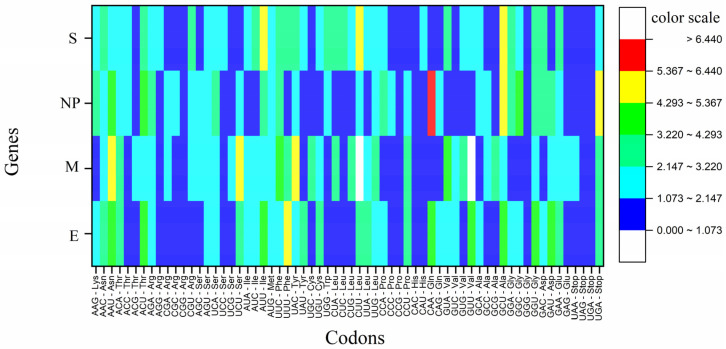
Heat map depicting the percent occurrence of codons in SARS-CoV-2 envisaged structural genes. Stop codons are included, and TGA is preferred over TAG and TAA. Color coding is given as a sidebar.

CGG is the rarest codon in the SARS-CoV-2 genome, and inserting two tandem CGG codons in the spike protein might result in ribosome pausing at rare codons. Ribosomal pausing has a role in the efficient regulation of protein expression and co-translational subdomain folding [93]. Codons CGG (Arg), CCG (Pro) and CAC (His) were rare in all the genes. GGG (Gly), CCC (Pro), and TCG (Ser) codons were rare in at least three genes (Figure 4). Codon CAA is highly used in NP (>5% of total codons) while used less than 1% in E and S genes.

For comparison among different strains of SARS-CoV-2 with delta strain, rare codon analysis was carried out considering all four structural genes as one sequence for each of the viral strains, and the number of rare codons was normalized. ACG, CAC, CCG, CGA, CGG, CGC, GCG, GGG, and TCG were rare in the delta and all other envisaged strains and had a frequency below 0.5%. Only the CGC codon frequency was slightly higher than 0.5% (0.62%) in Sarbacoviruses. We then performed pairwise comparisons between the codon frequencies and found no statistically significant difference. The analysis indicated that for all the VoCs, including delta and Sarbacoviruses, nine codons are rare. 

### 3.10. Codon Preference of SARS-CoV-2 Gene Delta Sequences Is towards Rare Human Isoacceptor tRNAs

It is suggested that suboptimal usage of isoacceptor host tRNAs helps slow and gradual translation of viral proteins to ensure correct folding [94]. Identification of the most preferred codons (for each amino acid) in the envisaged structural genes of the SARS-CoV-2 delta strain and the most abundant isoacceptor tRNAs in human cells revealed that only for ILeu codon, the preferred codon is matched with the respective most abundant isoacceptor tRNAs in human hosts (Table 6). Other than Ileu, out of 18 amino acids, only four amino acids (Phe, Leu, Ala, and Tyr) preferred codons were matched with abundant isoacceptor tRNA (in three genes out of four). The results suggested that the codons preferred by the envisaged genes of the delta strain of SARS-CoV-2 do not match the abundant tRNA pool in the human body (Table 6).

### 3.11. Vaccine Candidate Designing Using Information Generated in the Study

Viral fitness may be reduced by introducing rare codons for the virus [14], introducing the codons that are one substitution away from stop codons [95], and deoptimizing codon pairs [96]. Based on the analysis of envisaged genes, authors constructed three vaccine candidates (only the envisaged structural gene included), and those constructs were analyzed systemically for viral fitness. The first construct was based on the information that our sequences are TT and AA dinucleotide rich, and attenuation is correlated to an increase in TA content and a decrease in TpT and ApA dinucleotide [53]. Therefore we recoded three overrepresented TT-containing codons (CTT, GTT, and CTT codons) and replaced them with low-occurrence TA-containing codons (Table 7). While designing the second construct, we replaced abundant codons with rare codons common to the envisaged genes ((Codons CGG (Arg), CCG (Pro), CAC (His) GGG (Gly), CCC (Pro), and TCG (Ser) were introduced)). Finally, in the third construct, we disrupted preferred codon pairs (Table 7; ACT-, AAT-, TTT-, TTG- GTT-, GGA, and CTT- initiated; only 5’ codon was deoptimized from the favored codon pair). 

Van Leuven and colleagues [13] verified in phage 174 that the folding stability of the deoptimized codon mRNA is the best predictor of virus fitness, followed by CAI. Furthermore, in the experimentation of Groenke and colleagues, it was proved that with the lowest codon pair score, the highest virus attenuation is obtained [21]. Virus fitness by codon deoptimization is correlated to the amount of recoding performed, and codon deoptimization taking only one feature CAI (ignoring mRNA stability and codon pair score) doesn’t result in sufficient attenuation [13]. Thus, systemically, we used all three parameters to assess our construct’s fitness. mRNA stability was highest (folding energy −1801.30 kcal/mol) for the construct where rare codons were introduced, and it was even more negative than the native construct. Higher negative values exhibited higher virus fitness (though CAI was the least and CPS was the lowest, exhibiting attenuation). MFE was low for the construct recoded with TA ending codons (−1684.4 kcal/mol). The effect is likely owing to the mRNA destabilizing effect of TA [97]; however, in this construct, the CAI value was not much less, and the CPS score was also similar to that of the wild type. Disruption of favored codon pairs resulted in reduced protein expression (low CAI), low CPS and low mRNA stability (all three parameters we tested). Also, to construct three out of seven codons, only two codons have abundant corresponding isoacceptor tRNA (Table 6), and all remaining five isoaccptortRNA were suboptimal. Therefore from our analysis, construct three recoded where the favored codon pair is disrupted by introducing rare codons emerged as the most suitable candidate. In future studies, one may further incorporate changes to have better deoptimization. Here it is noteworthy that virus fitness is a complex term and results from many epistatic and genetic factors, which we ignored here due to the study limitations.

### 3.12. CpG Suppression in Different Constructs

Zinc finger antiviral protein (ZAP) powerfully restricts the viruses with elevated CpG and TpA dinucleotide frequencies [98,99], and the same is proved by knock-out experiments where attenuation in CpG- and UpA-high viruses was reversed in ZAP knock-out cell lines. CpG suppression in RNA and reverse transcribing viruses previously reported to be ZAP sensitive with odds ratio Sindbis virus (0.90), Semliki forest Virus (0.89), Venezuelan equine encephalitis virus (0.76), Ebolavirus (0.60), Hepatitis B virus (0.52), Moloney Murine Leukemia Virus (0.51), Marburg virus (0.53), Alphavirus M1 (0.89), Ross River Virus (0.82). In contrast, the odds ratio was less for ZAP-insensitive HIV-1 (0.21), the Yellow fever virus (0.38), and the Vesicular stomatitis virus (0.48) [98], suggesting that for higher odds ratios, the virus becomes ZAP-sensitive. For our constructs, the odds ratios were 0.268, 0.268, 0.635, and 0.63 for the wild-type delta construct and constructs 1, 2, and 3, respectively, with the highest odds ratio of 0.635 for construct 2 reported. It indicates the ZAP sensitivity of recoded constructed 2 and 3. The CpG suppression was highest in construct 2. 

An experiment of CpG enrichment from 02 CpGs to 39 CpGs in mutant L and 02 to 43 CpGs in LCG-HI in HIV-1 demonstrated ~100-fold lower replication than WT in primary lymphocytes [100]. In MEF-1 poliovirus, a type 2 wild poliovirus prototype strain with neurovirulence in humans, with the increasing substitutions, virus fitness was decreased but reduced most efficiently by increasing the frequencies of CpG and UpA dinucleotides [101]. The changes were brought in capsid region and CpG high constructs, namely ABc7 (80 CpG and 34 TpA) and ABc8 (90 CpG and 26 TpA), which exhibited a reduction in relative plaque area and relative plaque yields compared to reference construct having 28 CpG and 36 TpA. In the Influenza virus, smaller plaque sizes in CpG-high and TpA-high mutants were observed than in WT or permuted virus that brought no changes in overall A/T composition [102]. In the present study, in constructs 2 and 3, the CpG content was increased from 98 (for native delta construct) to 237 and 227, respectively, so we may expect the reduction of expression in our recoded constructs also. In the E7 genome, two segments were taken for the study, contributing to 16.7% and 14.2% of the full-length genome. CpG or TpA dinucleotides were altered from both regions. It was possible to reduce the CpG and TpA frequencies to approximately one-third or to enhance to 2.5–3-fold the wild-type levels in a gene sequence. The infectivity of permuted control sequence was similar to that of the wild type. CpG high in both segments resulted in viral output approximately 7000-fold lower, while TpA high in both segments had approximately 30-fold lower viral output after 24 h. This means the attenuation was higher for CpG enhancement than for TpA enhancement [40]. CpG and TpA alteration with their impact on virus replication has been given in Table 8. The same concord with our results, where we found introducing rare codon and codon pair disruption more effective than enhancing TpA content. 

Regarding the role of spacing between CpGs, it is demonstrated that when CpG is present in pairs, the DC stimulation is enhanced, and CD8 T cells are highly activated [103]. In the present study, in the native construct, no CpG dimer was present, while 04, 09, and 06 CpG dimers were present in constructs 1, 2, and 3. More CpGs in the sequence lead to increased IL10 and IL12 secretion [103]; thus highest IL10 and IL12 secretion will be there with construct 2.

**Table 8:** Impact of CpG and TpA enhancements and genomic compositions on different viruses.

Name of Virus	Virus Type/Name Assigned	%GC	CpG	TpA	∆CpG	∆TpA	Impact of CpG and TpA Enhancement	Reference
HIV-1	WT	*	02	--	--	--	High replicative fitness	[100]
	L	*	39	--	37	--	~100-fold lower levels than HIV-1 WT	
	L_CG-HI_	*	43	--	41	--		
Influenza A virus	Wild type	46	28	43	--	--	High replicative fitness	[102]
	CpG high	46	114	45	+86	+2	10–100 fold reduced viral loads in the lungs of mice infected with 200PFU and substantially greater attenuation of pathogenicity	
	TpA high	46	29	116	+1	+73	10–100 fold reduced viral loads in lungs of mice infected with 200PFU	
Polio virus Capsid Region	Wild	47.1	28	36	--	--	High replicative fitness	[101]
	ABC7	53.3	80	34	52	−2	Relative plaque area is 0.651, and relative plaque yield is 0.72 at 37 °C	
	ABC8	59.3	133	29	105	−7	Relative plaque area is 0.549, and relative plaque yield is 0.36 at 37 °C	
Zika	Wild	49.8	60	43	--	--	Lethal to mice	[104]
	Permuted	49.8	60	43	0	0	Lethal to mice	
	E+32CpG	49.9	92	42	32	−1	Replication not reduced	
	E+102CpG	49.9	162	43	102	0	Reduced replication in VERO and RD cells lines	
	E/NS1-176CpG	49.9	236	43	176	0	Reduced replication in VERO and RD cells lines	
Dengue virus type 2	Wild-type E	*	20	55	--	--	Increased frequencies of CpG and TpA attenuated the virus to degrees proportional to the numbers of additional CpG and UpA dinucleotides incorporated	[105]
	E recoded	*	87	86	67	31		
	Wild Type NS3	*	32	68	12	13		
	NS3 recoded	*	99	111	79	56		
	Wild type NS5	*	62	91	42	36		
	NS5 recoded	*	147	134	127	79		
E7 virus segment 1	Native (W)	47.6%	--	--	−51	−62	High replicative fitness	[40]
	Permuted (P)	47.6%	51	62	--	--		
	CpG-zero (c)	44.3%	0	70	51	8	A 100-fold increase in relative luminescence as early as 4 h post-transfection in E7 replicon having a luciferase gene that replaces structural genes	
	UpA-low (u)	50.9%	56	19	5	−43		
	Both-low (cu)	47.5%	0	19	−51	−43	10-fold enhancements in replication, two-fold greater resistance to IFNβ than WT	
	CpG-high (C)	56.5%	180	52	129	−10	100- to 10,000-fold impairments in replication # C/W has 144-fold less replication # U|W has 10 fold greater amplification	
	UpA-high (U)	40.9%	38	171	−12	109		
E7 virus segment 2	Native (W)	47.1%	--	−18	--	−48	High replicative fitness	
	Permuted (P)	47.6%	18	48	0	0		
	CpG-zero (c)	45.5%	0	48	−18	0	6-fold increase in relative luminescence as early as 4 h post-transfection in E7 replicon having a luciferase gene that replaces structural genes	
	UpA-low (u)	50.0%	21	14	3	−34		
	Both-low (cu)	48.5%	0	38	−18	−10	10-fold enhancements in replication, two-fold greater resistance to IFNβ than WT	
	CpG-high (C)	56.4%	135	38	116	−10	100- to 10,000-fold impairments, two-fold greater susceptibility to IFNβ # W|C has1500-fold less replication # WU like UU	
	UpA-high (U)	39.2%	15	151	−3	103		

* Not provided in MS. # When one segment is modified, and one segment is wild type (W).

Compositional analysis of each construct revealed that in native and construct 1, where TpT was converted to TpA ending codons, as expected, T/A composition was the same (60.2%). While for constructs 2 and 3, overall AT composition was less than the native construct, 56.11% and 54.59%, respectively. We have examples of viruses like IAV [102] or Zika [104] where, despite no changes in overall genomic AT/GC composition, enhancement in CpG and TpA, attenuated virus. On the other hand, in polio [101] and the E7 virus [40], CpG and TpA alterations with genome composition changes attenuated viruses, suggestive of a role of CpG and TpA in virus attenuation rather than composition.

## 4. Discussion

The SARS-CoV-2 delta strain has caused millions of death and has already proven to be one of the significant variants of concern. To counter the virus, scientists worldwide are continuously working to develop efficacious vaccine candidates. Since vaccine candidate development is a time-consuming process, if preliminary studies are done in-silico, it will save much time and resources. In the present study, we envisaged different molecular features of four structural genes, E, M, NP and S, from the perceptive of vaccine candidate development. The analysis is helpful in incorporating essential features during designing through the synthetic biology approach, and later, the viable attenuated virus can be rescued by using the reverse genetics approach. 

*Pasteurella multocida* is an avian cholera pathogen, and to construct an attenuated vaccine candidate, it was cultured from 37° to 45°. Among many descendants, one developed with low virulence and high immunogenicity [106]. In the experiment of Xia and colleagues [107], genomic features, including the GC content and dinucleotide frequencies, were envisaged to identify possible reasons behind thermal attenuation. In the attenuated low pathogenicity strain, the GC content was low despite the fact that more GC content would enhance thermal stability during raised temperature, and GC-rich codons encoded amino acids alanine and arginine would impart in thermal stability of the proteins. Investigation of other attenuated viruses revealed that without altering overall genomic AT/GC composition, only enhancement in CpG and TpA content attenuated viruses like Zika [104] and IAV [102]. Contrarily, in the polio [101] and E7 viruses [40], CpG and TpA alterations with genome composition changes attenuated the viruses, suggesting that the CpG and TpA have a more significant role in attenuation than composition. 

Selection for disfavoured codon pairs leads to unintended increases in CpG and UpA dinucleotide frequencies that also attenuate replication. In the viral genomes, CpG and UpA dinucleotides are present at low frequencies. Tulloch and colleagues manipulated a human gut virus, namely echovirus 7, where they made two sets of mutations. In one set, the codon pair frequencies were altered, keeping CpG and TpA constant. In contrast, in the second set, codon pair frequencies were kept the same while the CpG and TpA content was altered. The results revealed that alteration in codon pair frequency doesn’t alter the viral fitness, but an increase in CpG or TpA weakens the virus, and it is possibly attributed to the viruses being targeted readily by the host immune system post increase in CpG content and not due to altered virus fitness [19]. Considering all the facts together, we suggest that while constructing SARS-CoV-2 vaccine candidates through synthetic biology approach, CG or TA content should be optimized in a way that CG content should neither be that low for the virus that helps in escaping the host defense system nor should be too high that before eliciting sufficient immune response it is eliminated by the immune system.

Furthermore, the ApA, TpA, and TpT dinucleotides were higher, and those of ApT, GpC, and CpG dinucleotides were lower in the vaccine strain of the *P. multocida* strain than in the virulent strain. In the structural genes of the delta strain of SARS-CoV-2, TpT, ApA, CpT and TpG were overrepresented, while GpG, CpC, GpA, and CpG dinucleotides were underrepresented.

While constructing the vaccine candidate with the synthetic biology approach, knowing how much nucleotide content needs to be changed to get attenuation is essential. In echovirus 7, ten-fold or greater attenuation in cell culture was achieved by replacing >12–15% of the genome with codon pair deoptimized sequences that typically increased the frequencies of CpG and UpA from 0.4–0.6 to 1.4–1.6 (CpG) and from 0.5–0.8 to 1.1–1.4 (UpA) respectively [19].

Since TpA is commonly underrepresented in organisms [108,109], a further decrease in TpA content is helpful in attenuating viruses for developing vaccine candidates against animal viruses also. One example is the classical swine fever virus (CSFV), where the codon deoptimization technique was used in the glycoprotein E2-coding region of CSFV, where deoptimization increased TpA. Inoculation with this virus showed the animal’s survival and remained clinically normal [110], indicating efficacy as a vaccine candidate for animal use. On the other hand, the Minute virus of mice, a Parvovirus, exhibited no attenuation followed by increasing TpA and showed a similar replication pattern as of wild-type virus [111]. Thus the effect of the elevation of TpA has a virus-specific impact and needs to be tested for viruses separately.

Hence it is suggested that for constructing the SARS-CoV-2 vaccine candidate, the overall permissible change in a genome is 10–15%. It is noteworthy that not all the ORFs experience the same degree of CpG suppression. CpG suppression is least in the E gene and ORF10, and both use underrepresented codon pairs, and CpG usage is high compared to other ORFs [112]. Hence while deciding the CpG content, it is also essential to keep in mind the original CpG usage of individual ORFs. This observation will be relevant to future strategies for a rationally attenuated SARS-CoV-2 vaccine.

For codon deoptimization, one may have the choice of using non-optimal codons or codon pairs from the host or virus itself (mentioned in the above section). In the present study, we envisaged various parameters like preferred codons, preferred codon pairs, and rare codons that may be used to recode the virus genetic sequence and design a codon-deoptimized vaccine candidate. We analyzed both the preferred and rejected codon pairs for gene recoding. Codons CGG (Arg), CCG (Pro) and CAC (His) were rare in all the genes envisaged. GGG (Gly), CCC (Pro), and TCG (Ser) codons were rare in at least three genes. Codons ACG, CAC, CCG, CGA, CGG, CGC, GCG, GGG, and TCG were rare in the delta strain, and all other envisaged strains and had a frequency below 0.5% except for CGC having slightly higher (0.62%) in Sarbacoviruses. Judicial usage of these rare codons, along with their intelligent placements (like placements near the 5′ region) in the recoded virus, is expected to attain an attenuated phenotype with the ability to evoke an immune response.

Generally, it is considered that for obtaining an attenuated vaccine candidate, it is essential to incorporate deoptimized (rare) codons [113] or codon pairs [7]. But there are examples where attenuated vaccine candidates have been designed by using excess optimized codon pairs. For example, in the attempt to construct an attenuated vaccine candidate against Vesicular Stomatitis Virus (VSV) by computer-aided design, two recombinant versions were prepared. One version contained the excess underrepresented codon pairs (L1Min), and the other one contained excess overrepresented codon pairs (L1sdmax), where all the manipulations were carried out into the polymerase gene ‘L.’ Multistep growth kinetics and plaque phenotypes of the wild type and the engineered one revealed that the L1sdmax version was both immunogenic and attenuated. This attenuation was not host range specific since it generated small plaques in all the cell lines tested [114]. This observation is likely attributed to overrepresented codon pairs altering the translation rate, leading to disrupted coordination between translation and protein folding. Here it is important to note that CpG is more effective in attenuation than TpA. The evidence is from the Influenza A virus, where both CpG and TpA high viruses were attenuated with 10–100 fold reductions in the viral loads in the lungs of infected mice. However, the pathogenicity of CpG-high viruses was substantially reduced [102]. The E7 virus was modified using 02 segments representing 16.7% and 14.2% of the full-length genome. When both segments were replaced with CpG high or TpA high segments, 100- to 10,000-fold impairments in replication were observed. However, out of two segments, if only one segment was CpG or TpA high and the second segment was WT, then CpG high/WT combination exhibited 144-fold less replication. Contrarily, TpA high/WT exhibited 10 folds greater amplification [40]. All the points indicate the more significant role of CpG in attenuation than TpA.

ACT-, AAT-, TTT- and TTG-initiated codons (Threonine, Asparagine, Phenylalanine and leucine) were preferred in at least 3 genes out of the four envisaged, whereas GTT-, GGA and CTT- initiated (Val, Gly and Leu) codons were preferred in at least two genes. In the E gene, Phenylalanine-, Leucine-, Serine-, and Tyrosine-initiated codons are preferred in all genes. In the delta, Valine-initiated codons were also preferred, while in other VoCs and Sarbecoviruses, Serine initiated (06) codon pairs were preferred. Phenylalanine and Leucine-initiated codon pairs were preferred for the M gene in all envisaged strains. In the N gene and S genes, Glycine-initiated codon pairs were preferred in all VoCs, including delta strain apart from Sarbecoviruses. Since codon preference is similar for all viruses, similarity in codon pair preferences is also expected, and as expected, many of the codon pair usages are the same for delta compared to other strains; however, some unique patterns were also present, which could be molecular signatures. Authors suggest investigators use the information where highly preferred codon pairs are initiated with specific codons for recoding viruses through excessive codon optimization or deoptimization.

Since the genetic code is redundant, 18 out of 20 amino acids are encoded by two, three, four, or six synonymous codons. The observed usage of these codons is different from what is expected and called codon bias. This bias can be species-specific and correlated with the tRNA pool. Together the tRNA pool and codon usage determine how efficiently a protein will be translated. Since the virus depends on host cell cellular machinery for protein translation, many viral genomes contain more host-preferred codons. In an elegant work of Chen et al.,(2020), it was demonstrated that if host codon usage is similar to that of viral codon usage, it reduces the burden on host translation machinery while increasing viral gene expression. Human genes, which have a similar codon usage pattern to viral genes, are upregulated during infection between the host and virus is very similar for symptomatic hosts than natural hosts [115], suggesting more severe outcomes of having high codon usage similarities between the host and the pathogen. SARS-CoV-2 delta strain virus also preferred codons; for them, fewer isoacceptor tRNAs were present, and it appears to be a strategy where, in the key structural motifs, the pace of translation is kept low to facilitate proper folding of viral protein. Our result corresponds to the results found in the Nipah Virus by Khandia et al. [74], where a suboptimal tRNA pool was used for encoding viral genes. Similarly, in the hepatitis A virus (HAV), which presents a highly biased codon usage as opposed to the host codon usage and usage of inefficient IRES, the virus is able to synthesize its proteins owing to the usage of less abundant tRNA pool of host that results in a poor replication rate, and thus it is difficult to culture virus in cell culture [116]. The attempt to make the tRNA pool more available to the HAV virus, in fact, resulted in a loss of fitness and which later recovered through a re-deoptimization [117]. Based on our study, we concluded that while constructing attenuated vaccine candidates through synthetic biology approach using structural genes, disruption of favored codon pairs is a better strategy compared to incorporating “one to stop” TA dinucleotide or incorporating rare codons. Further, through reverse genetics, the desired deoptimized recombinant recoded virus may be rescued from cell culture and used to investigate efficacy and protection in the future.

## 5. Conclusions

Virus recoding, taking advantage of the synthetic biology approach, is an emerging technique in constructing vaccine candidates. Changing the overall nucleotide composition, CpG and TpA content, codon or codon pair deoptimization or excessive optimization, and knowledge related to the host tRNA pool are a few strategies currently being adapted for attenuating pathogens for vaccine candidate development. In the present study, we envisaged various molecular features of four structural genes of the SARS-CoV-2 virus delta strain. The study’s outcome encompasses information relating to the overall composition, where we found the genome rich in A/T nucleotides, specifically in T nucleotides. The information related to dinucleotide proportion may be used to carefully recode the virus so that its CpG content remains in a way that it will not escape the immune response.

On the other hand, it should not be too high to be removed instantly by the immune system. Codons CGG (Arg), CCG (Pro) and CAC (His) are rare in all the envisaged genes, while most of them preferred ACT-, AAT-, TTT-, and TTG-initiated codons. We also envisaged a positive codon context in S gene. The information related to the human isoacceptor tRNA pool and preferred codons in delta virus also might be helpful while considering the codons while recoding. Overall the information generated in the present study will be beneficial for researchers who are considering synthetic biology approach to develop a vaccine candidate again the deadly SARS-CoV-2 strain, and they may choose to have various options in combination to achieve a safe and efficacious vaccine candidate. Instead of incorporating rare codons, disruption of favored codon pairs is a more viable strategy in obtaining better vaccine candidates owing to both reduced protein expression and lower transcript stability.

## Figures and Tables

**Table 1:** Average nucleotide composition of structural genes of SARS-CoV-2 delta strain.

		%A	%C	%T	%G	%G+C	%A+T	%A3	%C3	%T3	%G3	%G3+C3	%A3+T3
E	Average	21.56	19.43	40.48	18.52	37.96	62.04	22.18	18.49	43.06	16.27	34.76	65.24
	SD	0.56	0.74	0.33	0.35	0.71	0.71	2.60	0.73	4.00	1.18	1.54	1.54
M	Average	25.56	21.93	31.74	20.78	42.70	57.30	24.22	22.85	36.79	16.14	38.99	61.01
	SD	0.03	0.08	0.08	0.02	0.09	0.09	0.06	0.10	0.12	0.05	0.12	0.12
NP	Average	31.75	25.01	21.25	21.98	47.00	53.00	30.58	22.58	31.72	15.12	37.70	62.30
	SD	0.09	0.06	0.09	0.09	0.08	0.08	0.20	0.09	0.19	0.12	0.16	0.16
S	Average	29.46	18.84	33.26	18.44	37.27	62.73	27.03	15.88	46.37	10.71	26.59	73.41
		0.04	0.05	0.03	0.04	0.05	0.05	0.05	0.09	0.11	0.06	0.13	0.13

**Table 2:** The most preferred codon among synonymous codons in structural genes.

S.No.	Single Letter Amino Acid	Codon	S	NP	M	E
1	F	TTT	**1.532**	0.462	0.909	0.8
		TTC	0.468	**1.538**	**1.091**	**1.2**
2	L	TTA	1.585	0.444	0.686	0.429
		TTG	1.132	**2**	0.686	0.857
		CTT	**2.038**	1.778	**2.057**	**3**
		CTC	0.623	0.444	1.029	0
		CTA	0.509	0.667	0.857	0.857
		CTG	0.113	0.667	0.686	0.857
3	I	ATT	**1.737**	**1.929**	**1.737**	**1**
		ATC	0.553	0.857	0.789	**1**
		ATA	0.711	0.214	0.474	**1**
4	V	GTT	**2.021**	1	1	**2.154**
		GTC	0.825	**1.5**	0	0.308
		GTA	0.619	0.5	**2**	0.923
		GTG	0.536	1	1	0.615
5	S	TCT	**2.242**	1.297	0.8	**3**
		TCC	0.727	0.486	1.2	0
		TCA	1.576	**1.459**	1.2	0.75
		TCG	0.121	0.324	0.4	0.75
		AGT	1.03	**1.459**	**1.6**	0.75
		AGC	0.303	0.973	0.8	0.75
6	P	CCT	**1.965**	1.143	0.8	**4**
		CCC	0.281	1	0	0
		CCA	1.754	**1.571**	**2.4**	0
		CCG	0	0.286	0.8	0
7	T	ACT	1.853	**2**	**1.429**	1
		ACC	0.421	0.75	1.143	0
		ACA	**1.6**	1	0.857	**2**
		ACG	0.126	0.25	0.571	1
8	A	GCT	**2.127**	**2.054**	**2.526**	1
		GCC	0.405	0.757	0.421	1
		GCA	1.367	0.865	0.842	0
		GCG	0.101	0.324	0.211	**2**
9	Y	TAT	**1.481**	0.5	0.889	0
		TAC	0.519	**1.5**	**1.111**	**2**
10	H	CAT	**1.529**	**1.5**	**1.6**	0
		CAC	0.471	0.5	0.4	0
11	Q	CAA	**1.484**	**1.543**	**1**	0
		CAG	0.516	0.457	**1**	0
12	N	AAT	**1.236**	**1.455**	0.727	**1.6**
		AAC	0.764	0.545	**1.273**	0.4
13	K	AAA	**1.258**	**1.375**	**1.143**	**2**
		AAG	0.742	0.625	0.857	0
14	D	GAT	**1.377**	**1.182**	0.333	**2**
		GAC	0.623	0.818	**1.667**	0
15	E	GAA	**1.447**	**1.333**	**1.714**	**1**
		GAG	0.553	0.667	0.286	**1**
16	C	TGT	**1.4**	0	**2**	0.667
		TGC	0.6	0	0	**1.333**
17	R	CGT	1.364	**1.333**	**2.143**	**2**
		CGC	0.136	1.111	0.857	0
		CGA	0	1.111	0.429	**2**
		CGG	0.409	0.444	0	0
		AGA	**2.727**	2	1.286	**2**
		AGG	1.364	0	1.286	0
18	G	GGT	**2.265**	0.909	1.429	**4**
		GGC	0.723	**1.545**	0.857	0
		GGA	0.867	1.182	**1.714**	0
		GGG	0.145	0.364	0	0

The most preferred codon among synonymous codons is given in bold.

**Table 3:** Average RSCU values for representative SARS-CoV-2 VoCs and Sarbecoviruses. The most preferred codon for an amino acid from the synonymous codon family is given in bold.

Codons	Amino Acid	Alpha	Beta	Gamma	Omicron	Sarbecoviruses	Delta
TTT	F	**1.010**	0.927	0.929	0.931	0.944	0.926
TTC		0.991	**1.073**	**1.071**	**1.069**	**1.056**	**1.074**
TTA	L	0.782	0.768	0.782	0.782	0.781	0.786
TTG		1.166	1.157	1.166	1.166	1.089	1.169
CTT		**2.214**	**2.245**	**2.200**	**2.214**	**2.082**	**2.218**
CTC		0.537	0.540	0.537	0.537	0.588	0.524
CTA		0.722	0.710	0.722	0.722	0.801	0.723
CTG		0.581	0.581	0.595	0.581	0.660	0.581
ATT	I	**1.616**	**1.597**	**1.583**	**1.609**	**1.634**	**1.601**
ATC		0.799	0.813	0.826	0.796	0.745	0.800
ATA		0.585	0.590	0.591	0.595	0.621	0.600
GTT	V	**1.533**	**1.528**	**1.528**	**1.533**	**1.473**	**1.544**
GTC		0.669	0.666	0.671	0.669	0.838	0.658
GTA		1.011	1.019	1.012	1.011	0.875	1.011
GTG		0.788	0.787	0.789	0.788	0.813	0.788
TCT	S	**1.841**	**1.835**	**1.879**	**1.835**	**1.770**	**1.835**
TCC		0.605	0.603	0.600	0.603	0.518	0.603
TCA		1.250	1.246	1.239	1.246	1.424	1.246
TCG		0.399	0.399	0.398	0.399	0.474	0.399
AGT		1.182	1.210	1.179	1.210	1.071	1.210
AGC		0.723	0.707	0.705	0.707	0.743	0.707
CCT	P	**1.986**	**1.986**	**1.988**	**1.977**	**1.787**	**1.977**
CCC		0.319	0.319	0.330	0.320	0.400	0.320
CCA		1.424	1.424	1.409	1.431	1.529	1.431
CCG		0.272	0.272	0.274	0.272	0.284	0.272
ACT	T	**1.562**	**1.572**	**1.583**	**1.559**	**1.523**	**1.571**
ACC		0.538	0.527	0.512	0.581	0.496	0.579
ACA		1.401	1.401	1.398	1.373	1.505	1.364
ACG		0.499	0.499	0.508	0.488	0.477	0.487
GCT	A	**1.927**	**1.934**	**1.927**	**1.927**	**1.920**	**1.927**
GCC		0.646	0.647	0.646	0.646	0.700	0.646
GCA		0.769	0.760	0.769	0.769	0.718	0.769
GCG		0.659	0.660	0.659	0.659	0.662	0.659
TAT	Y	0.684	0.686	0.691	0.718	0.626	0.718
TAC		**1.317**	**1.314**	**1.310**	**1.283**	**1.374**	**1.283**
CAT	H	**1.157**	**1.164**	**1.150**	**1.157**	**0.938**	**1.157**
CAC		0.343	0.336	0.350	0.343	0.562	0.343
CAA	Q	**1.007**	**1.013**	**1.007**	**1.007**	**0.977**	**1.007**
CAG		0.493	0.487	0.493	0.493	0.523	0.493
AAT	N	**1.252**	**1.252**	**1.247**	**1.255**	**1.198**	**1.255**
AAC		0.748	0.748	0.753	0.746	0.802	0.746
AAA	K	**1.436**	**1.444**	**1.449**	**1.439**	**1.462**	**1.444**
AAG		0.564	0.556	0.551	0.561	0.538	0.556
GAT	D	**1.219**	**1.214**	**1.217**	**1.223**	**1.158**	**1.223**
GAC		0.781	0.786	0.783	0.777	0.842	0.777
GAA	E	**1.366**	**1.363**	**1.363**	**1.381**	**1.236**	**1.374**
GAG		0.634	0.637	0.637	0.619	0.764	0.627
TGT	C	**1.021**	**1.017**	**1.017**	**1.517**	**0.885**	**1.017**
TGC		0.480	0.483	0.483	0.483	0.615	0.483
CGT	R	1.660	1.675	1.665	1.698	1.606	1.710
CGC		0.508	0.509	0.501	0.516	0.722	0.526
CGA		0.866	0.866	0.957	0.875	1.061	0.885
CGG		0.208	0.140	0.173	0.210	0.156	0.213
AGA		**2.037**	**2.071**	**2.053**	**2.039**	**1.652**	**2.003**
AGG		0.722	0.739	0.651	0.663	0.803	0.663
GGT	G	**2.168**	**2.173**	**2.174**	**2.138**	**1.882**	**2.151**
GGC		0.767	0.765	0.776	0.788	0.765	0.781
GGA		0.936	0.933	0.919	0.945	1.161	0.941
GGG		0.129	0.129	0.132	0.129	0.191	0.127

**Table 4:** Percent frequency of top 20 high occurrence codon pairs.

Gene Name	Envelope		Nucleocapsid		Membrane		Spike	
% frequency of top 20 codon pairs								
1.	TTA-ATA	1.46	CAA-CAA	0.96	ATT-GCT	1.79	GTT-TAT	0.54
2.	TCG-GAA	1.46	AAA-GAT	0.95	TGT-CTT	0.89	GGT-GTT	0.50
3.	TAC-TCA	1.46	ATT-GGC	0.72	GGA-GCT	0.89	TTT-GGT	0.47
4.	GTT-TCG	1.46	AAG-AAG	0.72	CTT-GTA	0.89	ACT-AAT	0.45
5.	GTT-AAT	1.46	CAA-GGA	0.72	CTT-CTA	0.89	GGT-GAT	0.40
6.	GTA-CTT	1.46	TCA-ACT	0.71	CTT-CGT	0.89	TTT-AAT	0.39
7.	GGT-ACG	1.46	CCT-GCT	0.71	ATG-TGG	0.89	TCT-AAC	0.39
8.	GAA-GAG	1.46	AGC-AGT	0.68	ACT-ATT	0.89	AAT-CTT	0.39
9.	CTT-TTT	1.46	GGA-ACT	0.61	GCT-TGT	0.88	AAT-GGT	0.38
10.	CTT-CTT	1.46	CAA-ATT	0.48	GAA-GAG	0.46	AAT-TTT	0.32
11.	ATG-TAC	1.46	ACT-CAA	0.48	ATA-ATT	0.46	AAC-AAA	0.32
12.	ATA-GTT	1.46	TTG-GAT	0.48	TTT-TTG	0.45	AAT-GTT	0.32
13.	AGC-GTA	1.46	TTG-CTG	0.48	TTG-CTT	0.45	GTT-TTT	0.32
14.	ACG-TTA	1.46	TAC-TAC	0.48	TGG-ATT	0.45	TAT-TCT	0.31
15.	AAT-AGC	1.46	GGC-AGT	0.48	CTC-CTT	0.45	GTT-GCT	0.31
16.	TTT-CTT	1.44	GGA-CCC	0.48	ATT-ACC	0.45	GCA-CAA	0.31
17.	TTG-CTA	1.44	GCT-GCT	0.48	AAT-ATT	0.45	ACT-TCT	0.31
18.	TTC-TTG	1.44	GAC-AAA	0.48	TTT-GCT	0.45	TAT-AAT	0.31
19.	TTC-GTG	1.44	CGT-GGT	0.48	TTT-GCG	0.45	AAT-GAT	0.31
20.	GTT-ACA	1.44	CGC-ATT	0.48	TTT-GCC	0.45	GGT-TTT	0.31

**Table 5:** (A) Comparison of the E gene of the delta strain with other strains. (B) Comparison of the M gene of the delta strain with other strains. (C) Comparison of the N gene of the delta strain with other strains. (D) Comparison of the S gene of the delta strain with other strains. Dissimilar preferred codon pairs are depicted in bold and underlined.

**(A)**									
**Alpha**	**Delta**	**Beta**	**Delta**	**Gamma**	**Delta**	**Omicron**	**Delta**	**Sarbecoviruses**	**Delta**
FY	FY	FY	FY	FY	FY	FY	FY	YS	**FY**
FL	LC	FL	LC	FL	LC	FL	LC	**VY**	LC
LC	LL	LC	LL	LC	LL	LC	LL	**VK**	LL
LL	FL	LL	FL	LL	FL	LL	FL	LC	FL
FL	FV	FL	FV	FL	FV	FL	FV	LL	FV
FV	LI	FV	LI	FV	LI	FV	LI	FL	LI
FV	CA	FV	CA	FV	CA	FV	CA	FV	CA
LI	CC	LI	CC	LI	CC	LI	CC	LI	CC
CA	CN	CA	CN	CA	CN	CA	CN	CA	CN
CC	SF	CC	SF	CC	SF	CC	SF	CC	SF
CN	SR	CN	SR	CN	SR	CN	SR	CN	**SR**
SF	SE	SF	SE	SF	SE	SF	SE	**SS**	SE
**SS**	YC	**SS**	YC	**SS**	YC	**SS**	YC	SE	YC
SR	YS	SR	YS	SR	YS	SR	YS	SF	YS
SR	**YV**	SR	**YV**	SR	**YV**	SR	**YV**	YC	**YV**
SE	**VS**	SE	**VS**	SE	**VS**	SE	**VS**	VS	VS
SF	**VT**	SF	**VT**	SF	**VT**	SF	**VT**	**VP**	**VT**
YC	VN	YC	VN	YC	VN	YC	VN	VN	VN
YS	VN	YS	VN	YS	VN	YS	VN	VN	VN
YS	**VV**	YS	**VV**	YS	**VV**	YS	**VV**	VV	VV
**(B)**									
**Alpha**	**Delta**	**Beta**	**Delta**	**Gamma**	**Delta**	**Omicron**	**Delta**	**Sarbecoviruses**	**Delta**
IA	IA	IA	IA	IA	IA	IA	IA	IA	IA
CL	CL	CL	CL	CL	CL	CL	CL	CL	CL
GA	GA	GA	GA	GA	GA	GA	GA	GA	GA
AC	LV	AC	LV	AC	LV	LV	LV	LV	LV
LV	LL	LV	LL	LV	LL	LL	LL	LL	LL
LL	LR	LL	LR	LL	LR	LR	LR	LR	LR
LR	MW	LR	MW	LR	MW	MW	MW	MW	MW
MW	TI	MW	TI	MW	TI	TI	TI	TI	TI
TI	AC	TI	AC	TI	AC	FL	**AC**	FL	**AC**
FL	**EE**	FL	**EE**	FL	**EE**	**FV**	**EE**	**FV**	**EE**
**FV**	**II**	**FV**	**II**	**FV**	**II**	FA	**II**	FA	**II**
FA	FL	FA	FL	FA	FL	FA	FL	FA	FL
FA	LL	FA	LL	FA	LL	FA	LL	FA	LL
FA	**WI**	FA	**WI**	FA	**WI**	**FN**	**WI**	**FN**	**WI**
FL	LL	**LY**	LL	**LY**	LL	**LY**	LL	**LY**	LL
**LY**	**IT**	**LG**	**IT**	**LG**	**IT**	**LG**	**IT**	**LG**	**IT**
**LG**	**NI**	LL	**NI**	LL	**NI**	LL	**NI**	LL	**NI**
LL	FA	**LM**	FA	**LM**	FA	**LM**	FA	**LM**	FA
**LM**	FA	FL	FA	FL	FA	FL	FA	FL	FA
FL	FA	FL	FA	FL	FA	FL	FA	FL	FA
**(C)**									
**Alpha**	**Delta**	**Beta**	**Delta**	**Gamma**	**Delta**	**Omicron**	**Delta**	**Sarbecoviruses**	**Delta**
QQ	QQ	QQ	QQ	QQ	QQ	QQ	QQ	QQ	QQ
KD	KD	KD	KD	KD	KD	KD	KD	KK	KD
ST	IG	ST	IG	ST	IG	ST	IG	QG	IG
PA	KK	PA	KK	PA	KK	PA	KK	IG	KK
QG	QG	QG	QG	QG	QG	QG	QG	KD	QG
IG	ST	IG	ST	IG	ST	IG	ST	GT	ST
SS	PA	SS	PA	KK	PA	KK	PA	KK	PA
KK	SS	KK	SS	LD	**SS**	LD	**SS**	ST	**SS**
LD	GT	LD	GT	LL	GT	LL	GT	**DD**	GT
LL	**QI**	LL	**QI**	**FY**	**QI**	**FY**	**QI**	PA	QI
**FY**	**TQ**	**FY**	**TQ**	YY	**TQ**	YY	**TQ**	GP	**TQ**
YY	LD	YY	LD	**YK**	LD	**YK**	LD	DK	**LD**
**YK**	LL	**YK**	LL	**GK**	LL	**GK**	LL	RI	**LL**
**GK**	YY	**GK**	YY	**GQ**	YY	**GQ**	YY	**PK**	**YY**
**GQ**	GS	**GQ**	GS	GS	GS	GS	GS	QI	**GS**
GS	GP	GS	GP	GP	GP	GP	GP	**YK**	GP
GP	AA	GP	AA	GT	AA	GT	AA	**LP**	**AA**
GT	**DK**	GT	**DK**	AA	**DK**	AA	**DK**	RG	DK
AA	**RG**	AA	**RG**	AA	**RG**	AA	**RG**	**KG**	RG
AA	**RI**	AA	**RI**	**AN**	**RI**	**AN**	**RI**	**PQ**	RI
**(D)**									
**Alpha**	**Delta**	**Beta**	**Delta**	**Gamma**	**Delta**	**Omicron**	**Delta**	**Sarbecoviruses**	**Delta**
VY	VY	VY	VY	VY	VY	VY	VY	NF	VY
YS	YS	YS	YS	**AL**	YS	YS	YS	**YE**	**YS**
YN	YN	YN	YN	AQ	YN	YN	YN	VY	**YN**
VF	VF	VF	VF	FG	VF	VA	**VF**	VF	VF
VA	VA	VA	VA	FN	VA	**V**	VA	TS	**VA**
TS	TS	TS	TS	**GA**	TS	TS	TS	SN	TS
TN	TN	TN	TN	GD	TN	TN	TN	**SF**	**TN**
SN	SN	SN	SN	GF	SN	SN	SN	**PF**	SN
NL	**NV**	NV	NV	GV	**NV**	NL	**NV**	NV	NV
NG	NL	NG	**NL**	IA	**NL**	NG	NL	**LD**	**NL**
IA	**NK**	NF	**NK**	IA	**NK**	IA	**NK**	**IT**	**NK**
IA	NG	ND	NG	NF	**NG**	IA	NG	**IA**	**NG**
GV	**NF**	IA	NF	**SF**	NF	GV	**NF**	GV	NF
GF	**ND**	IA	ND	SN	**ND**	GF	**ND**	GD	**ND**
GD	GV	GV	GV	TN	GV	GD	GV	FN	GV
**GA**	GF	GF	GF	TS	GF	**GA**	GF	FG	**GF**
FN	GD	GD	GD	VA	GD	FN	GD	**DV**	GD
FG	FN	FN	FN	VF	FN	FG	FN	**DI**	FN
AQ	FG	FG	FG	YN	FG	AQ	FG	**AD**	FG
**AL**	AQ	AQ	AQ	YS	AQ	**AL**	AQ	**AA**	**AQ**

**Table 6:** Table for human tRNA isotype in human cells. The preferred codons corresponding to the most abundant tRNA pool are given the bold font in each row corresponding to each gene. * The amino acid is absent in a particular gene. The isoacceptor tRNAs used in disrupted codon pair construct are given in italics.

	tRNA Isotype in Human	Total Count	Most Preferred Codon			
			S	NP	M	E
**Phe (F)**	AAA(0), *GAA*(10)	10	TTT	**TTC**	**TTC**	**TTC**
**Leu (L)**	AAG(9), GAG(0), *CAG*(9),TAG(3), CAA(6), TAA(4)	31	**CTT**	TTG	**CTT**	**CTT**
**Ile (I)**	AAT(14), GAT(3), CAT(0), TAT(5)	22	**ATT**	**ATT**	**ATT**	**ATT** ATC ATA
**Val (V)**	AAC(9), GAC(0), CAC(11), *TAC*(5)	25	GTT	GTC	GTA	GTT
**Ser (S)**	AGA(9), GGA(0), CGA(4), TGA(4), ACT (8),GCT(8)	25	**TCT**	TCA AGT	AGT	**TCT**
**Pro (P)**	AGG(9), GGG(0), CGG(4), TGG(7)	20	**CCT**	CCA	CCA	**CCT**
**Thr (T)**	AGT(9),GGT(0), *CGT*(5), TGT(6)	20	ACA	**ACT**	**ACT**	ACA
**Ala (A)**	AGC(22), GGC(0), CGC(4), TGC(8)	34	**GCT**	**GCT**	**GCT**	GCG
**Tyr (Y)**	ATA(0), GTA(13),	13	TAT	**TAC**	**TAC**	**TAC**
**His (H)**	ATG(0), GTG(10)	10	CAT	CAT	CAT	*
**Gln (Q)**	CTG(13), TTG(6)	19	CAA	CAA	CAA CAG	*
**Asp (N)**	ATT(0), *GTT*(20)	20	AAT	AAT	**AAC**	AAT
**Lys (K)**	CTT(15), TTT(12)	27	AAA	AAA	AAA	AAA
**Asp (D)**	ATC(0), GTC(13)	13	GAT	GAT	**GAC**	GAT
**Glu (E)**	CTC(8), TTC(7)	15	GAA	GAA	GAA	GAA **GAG**
**Cys (C)**	ACA(0), GCA(29),	29	TGT	*	TGT	**TGC**
**Arg (R)**	ACG(7), GCG(0), *CCG*(4), TCG(6), CCT(5), TCT(6)	28	AGA	**CGT**	**CGT**	AGA CGA AGA
**Gly (G)**	ACC(0), GCC(14), CCC(5), TCC(9)	28	GGT	**GGC**	GGA	GGT

**Table 7:** Exhibition of codons recoded in the single construct having all four envisaged genes with intracodon and junctional CpG and TpA with O/E of CpG and TpA.

		From Codon	Frequency	To Codon	Frequency	CAI	Nc	CPS	MFE (kcal/mol)	%G+C	Intracodon CpG	CpG at p3-1 Unction	Total CpG	∆CpG	Intracodon TpA	TpA at p3-1 Unction	Total TpA	∆TpA	CpG-O/E	TpA-O/E
1.	Wild-type SARS-CoV-2 Delta strain	-	-	-	-	0.699	48.6	0.158	−1776.90	40.1	66	34	100	--	181	192	373	--	0.268	1.005
2.	Overrepresented codons to TA ending codons leading to TpT dimer to TpA (Construct 1)	CTT	31.6	CTA	10	0.666/659	45	0.158	−1684.40	39.98	66	34	100	0	421	144	565	192	0.268	1.386
		ATT	32.6	ATA	11.5															
		GTT	30.1	GTA	12.5															
3.	Introduction of rare codons (Construct 2)	CCT	19.5	CCG	1.5	0.558	43.4	0.143	−1801.30	43.89	194	43	237	137	181	149	330	−43	0.635	0.874
		CAT	10	CAC	3.5															
		CGT	10.5	CGG	2															
		GGT	32.1	GGG	3.5															
		CCA	19.5	CCC	1.5															
		TCT	25.1	TCG	3															
4.	Disruption of favored codon pairs at the 5′ end (Construct 3)	ACT	33.1	ACG	4	0.577	41	0.152	−1747.80	45.41	137	90	227	127	241	105	346	−27	0.63	0.938
		AAT	36.6	AAC	24.1															
		TTT	32.6	TTC	18.5															
		TTG	17.5	CTG	6															
		GTT	30.1	GTA	12.5															
		AGG	6.5	CGG	2															
		CTT	31.6	CTG	6															

## Data Availability

Provided in Supplementary Table S1.

## Supplementary Materials

The following supporting information can be downloaded at: https://www.mdpi.com/article/10.3390/vaccines11020487/s1, Table S1: The accession numbers of the sequences.
